# 299. Distinct TGF-β1 Patterns in Patients with Sepsis and Non-alcoholic Fatty Liver Disease

**DOI:** 10.1093/ofid/ofad500.371

**Published:** 2023-11-27

**Authors:** Neven Papic, Nina Vrsaljko, Leona Radmanic, Petra Simicic, Juraj Krznaric, Branimir Gjurasin, Snjezana Zidovec Lepej, Adriana Vince

**Affiliations:** University Hospital for Infectious Diseases Zagreb, Zagreb, Grad Zagreb, Croatia; University Hospital for Infectious Diseases Zagreb, Zagreb, Grad Zagreb, Croatia; University Hospital for Infectious Diseases Zagreb, Zagreb, Grad Zagreb, Croatia; University Hospital for Infectious Diseases Zagreb, Zagreb, Grad Zagreb, Croatia; School of Medicine, University of Zagreb, Zagreb, Grad Zagreb, Croatia; University Hospital for Infectious Diseases Zagreb, Zagreb, Grad Zagreb, Croatia; University Hospital for Infectious Diseases Zagreb, Zagreb, Grad Zagreb, Croatia; University Hospital for Infectious Diseases Zagreb, Zagreb, Grad Zagreb, Croatia

## Abstract

**Background:**

There is growing evidence that non-alcoholic fatty liver disease (NAFLD) might impact infection course and outcomes. However, the immune responses in patients with NAFLD and sepsis have not been described. TGF-β1 is a key biological regulator that exhibits broad immunosuppressive and healing effects in sepsis. Although TGF-β pathway dysregulation is described in patients with NAFLD, TGF-β responses in patients with sepsis and NAFLD are still unknown. The aim was to determine whether patients with NAFLD and sepsis have distinct circulating TGF-β1 patterns.

**Methods:**

A prospective cohort study included 80 patients with community-acquired sepsis (SOFA > 2); 40 patients with NAFLD and 40 without NAFLD. TGF-β1 serum concentrations were analyzed at admission and on day 5 of hospitalization. Routine demographic, laboratory, microbiological, and clinical data were collected. Data are presented as frequencies and medians with interquartile ranges.

**Results:**

The main sources of sepsis were respiratory (25, 31.2%), urinary (18, 22.5%), and skin and soft tissue infection (16, 20%). Groups were matched regarding age (65 [53-74] years), sex (39 males), and comorbidities, except for obesity which was more frequent in the NAFLD group (BMI of 35 [28-40] vs 26 [22-28] kg/m^2^). There were no differences in routine laboratory findings including CRP, procalcitonin, lactate, and WBC, except for GGT which was higher in the NAFLD group (60 [31-115] vs 33 [21-69] IU/L). Upon admission, patients with NAFLD had higher TGF-β1 concentrations than patients without NAFLD (207 [87-350] vs 65 [13-172] pg/mL). However, distinct temporal changes were observed on day 5; while TGF-β1 decreased in most patients with NAFLD (in 24, 60%, median of 140 [43-305] pg/mL), in the non-NAFLD group TGF-β1 concentrations increased in 34 (85%) patients to 151 [82-367] pg/mL), as shown in Figure 1.

**Figure:**
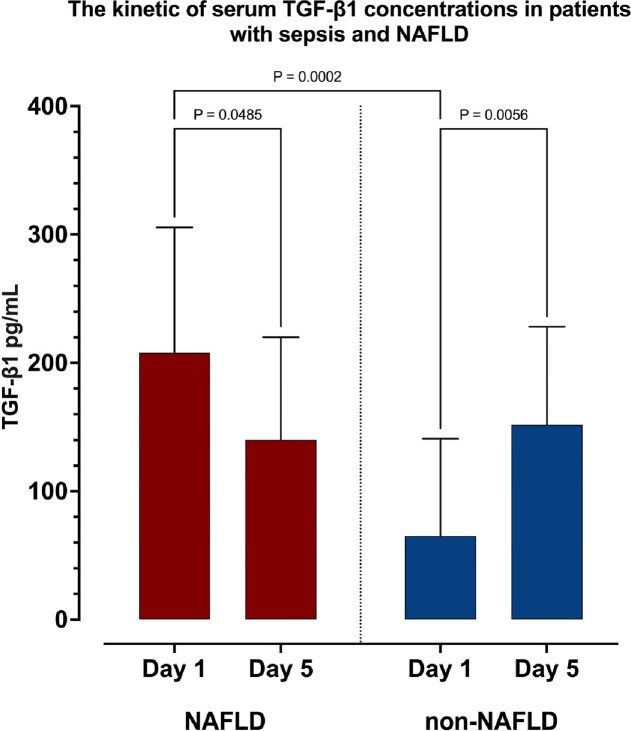

**Conclusion:**

Patients with NAFLD have higher baseline TGF-β1 serum concentrations that fail to further increase, but rather decrease in a compensatory anti-inflammatory phase of sepsis. A better understanding of these TGF-β1 changes could potentially help elucidate the complex and aberrant immune responses in NAFLD patients during sepsis.

**Disclosures:**

**All Authors**: No reported disclosures

